# Supplementary material for: Dysregulated Alternative Splicing in Breast Cancer Subtypes of RIF1 and Other Transcripts
Source: Int J Mol Sci. 2025 Jul 29;26(15):7308. doi: 10.3390/ijms26157308 (PMC12347697; doi:10.3390/ijms26157308)
Supplement: Supplementary file 1 [file ijms-26-07308-s001.zip › ijms-3748455-supplementary.pdf]

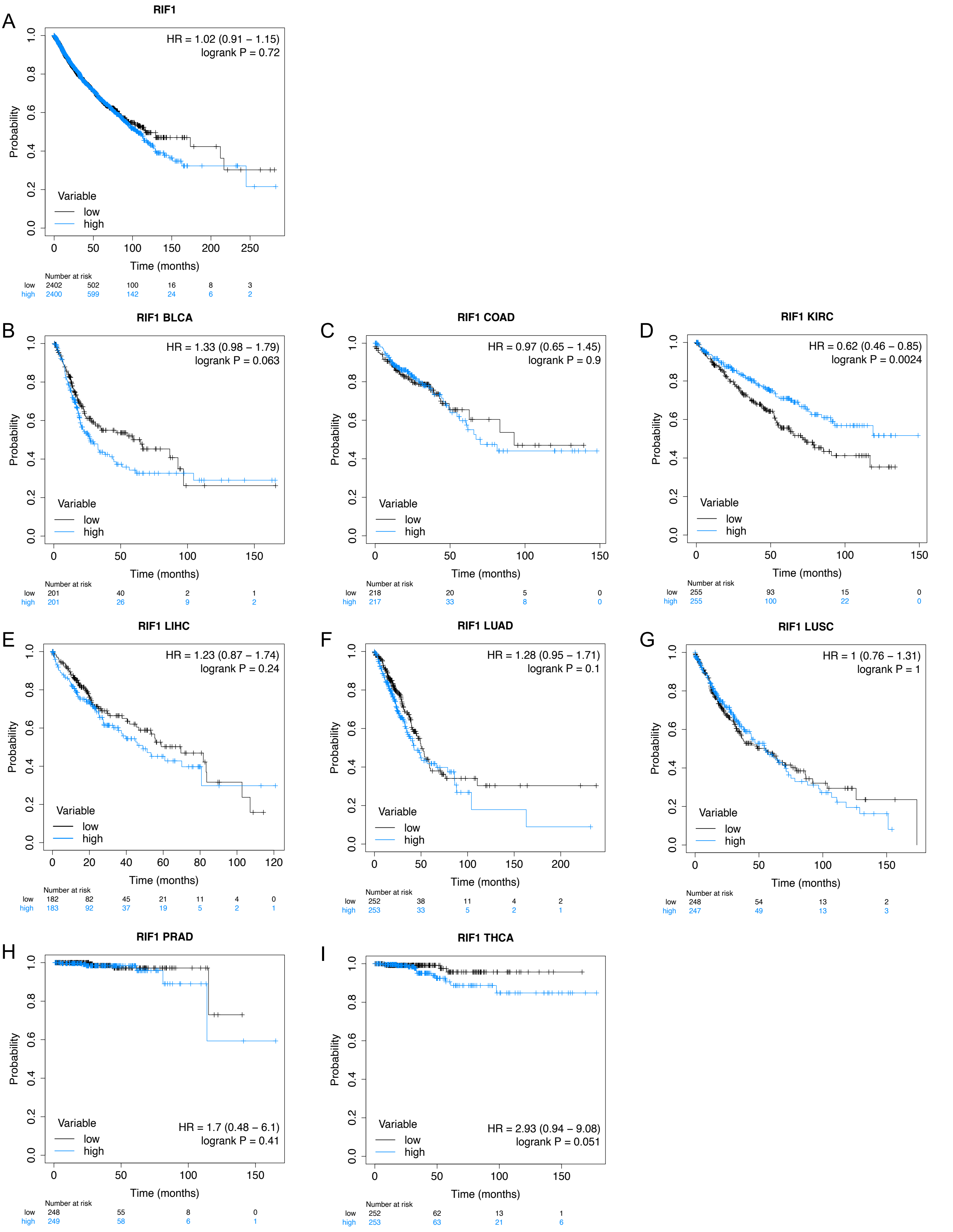

Figure S1. Kaplan-Meier survival analyses for RIF1 mRNA expression levels in different cancer types

Kaplan-Meier plots showing progression-free survival of patients with high (blue) and low (black) RIF1 mRNA expression in A) all cancer types combined, and (B-I) subdivided by cancer type. In each plot, patients were grouped based on median RIF1 mRNA expression.

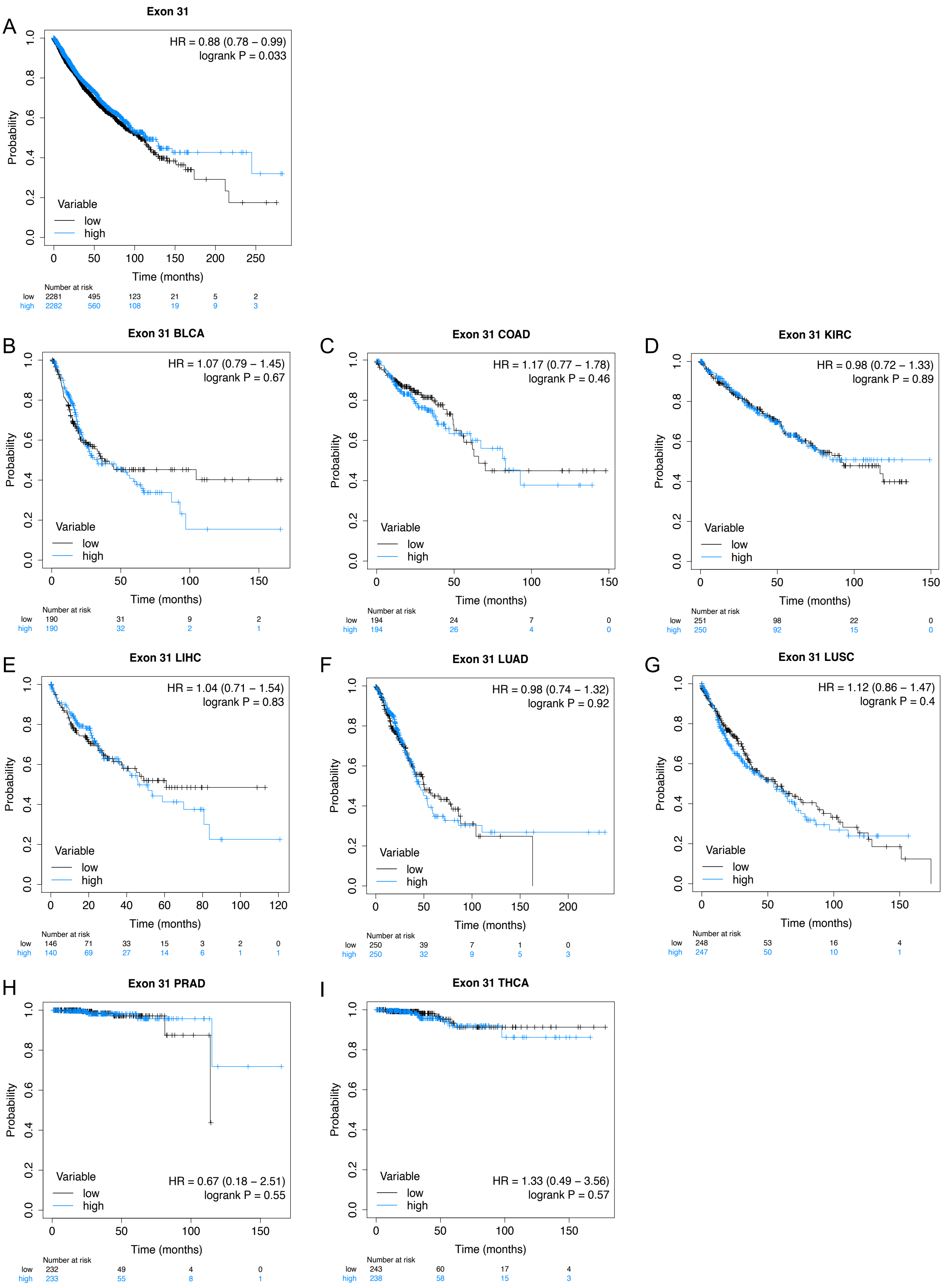

Figure S2. Kaplan-Meier survival analyses for RIF1 Exon 31 inclusion in different cancer types
Kaplan-Meier plots showing progression-free survival of patients with high (blue) and low (black) RIF1 Exon 31 inclusion (PSI) in A) all cancer types combined, and (B-I) subdivided by cancer type. Patients were grouped based on median RIF1 Exon 31 PSI value.

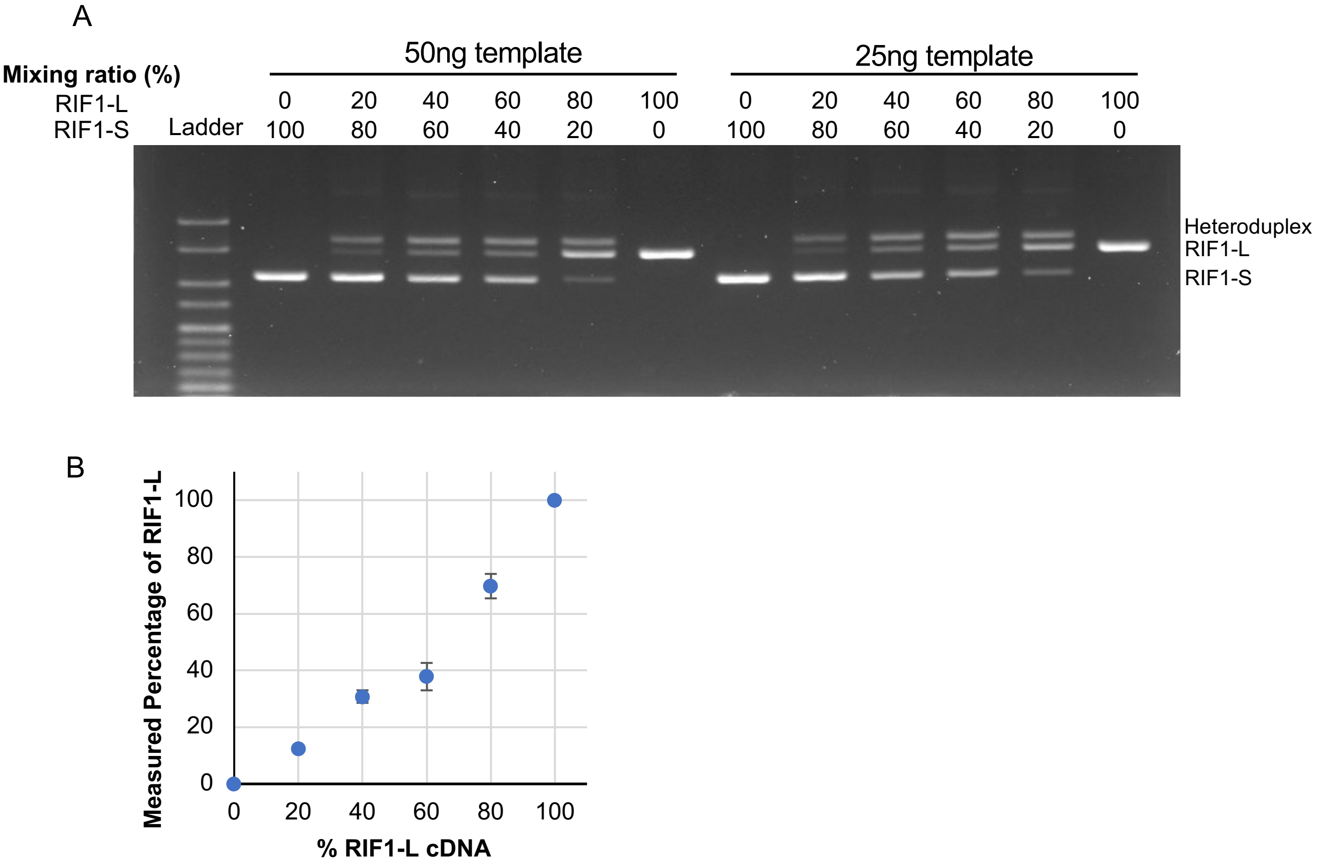

Figure S3. Detection of RIF1 isoforms by RT-PCR

A) Validation of competitive PCR for detection of relative amounts RIF1 variants by RT-PCR. Briefly, cDNA samples were prepared from cell lines expressing RIF1-L or RIF1-S only, then mixed at the indicated ratios before analysis by competitive RT-PCR using the primer pair described in Fig. 3A. Amplification conditions are as follows; one cycle of 98ºC for 5 minutes; 10 cycles of 98ºC for 5 seconds, 69ºC for 5 seconds, 72ºC for 20 seconds; followed by 30 cycles of 98ºC for 5 seconds, 64ºC for 5 seconds, 72ºC for 20 seconds. Image shows visualization of fragments corresponding to RIF1-L, RIF1-S and a heteroduplex of RIF1-L + RIF1-S after agarose gel separation. B) Quantification of relative RIF1-L measured by competitive PCR at various ratios of RIF1-L:RIF1-S cDNA. 50% of signal from the RIF1-L+RIF1-S heteroduplex band is assigned to RIF1-L and 50% to RIF1-S during quantification.

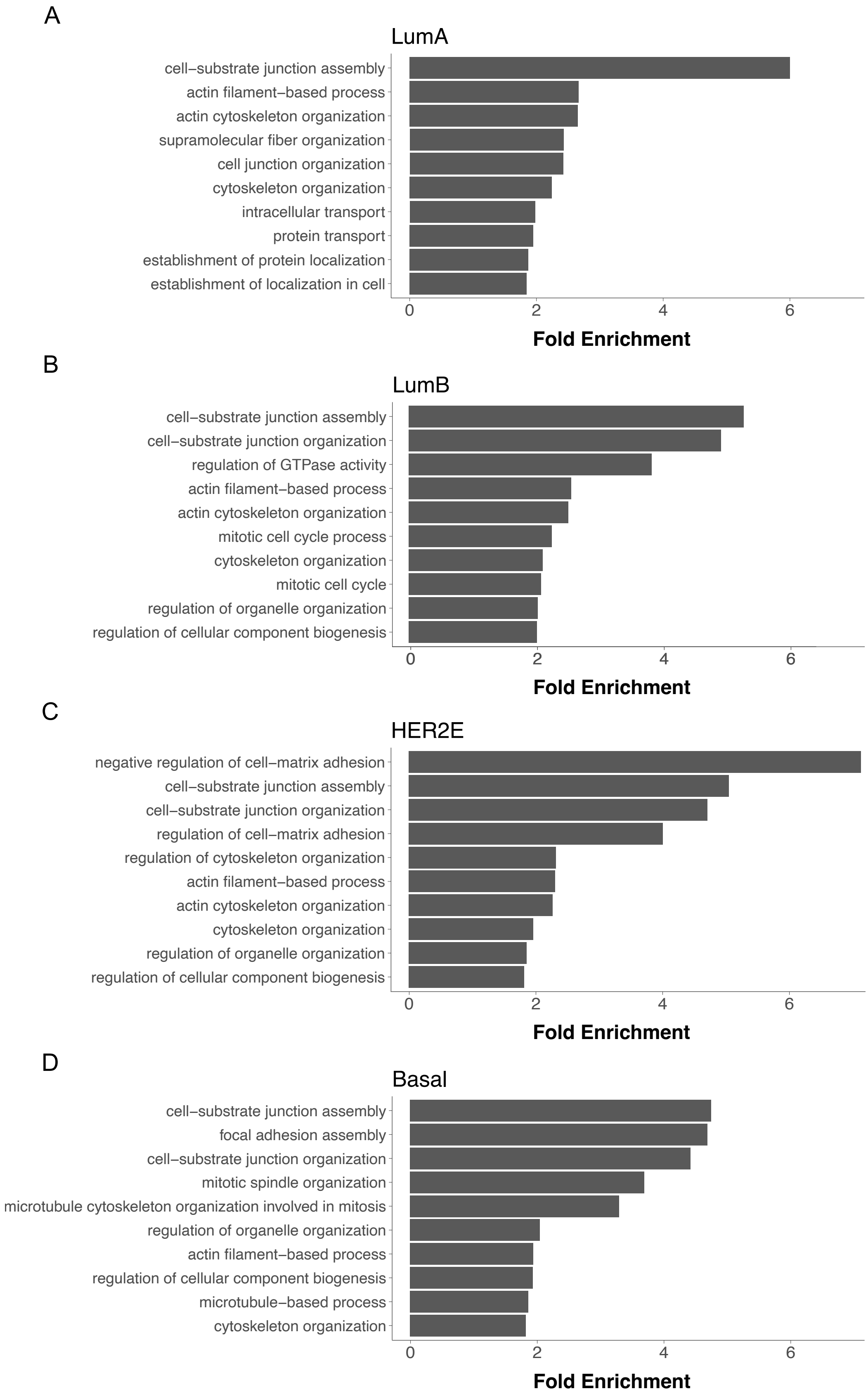

Figure S4. GO Enrichment for 'Cancer-Altered' Long and Short exons in breast cancer subtypes
Bar plots showing fold enrichment of the top 10 enriched Gene Ontology biological processes for genes containing 'Cancer-Altered' Long and Short exons in A) LumA, B) LumB, C) HER2E, and D) Basal breast cancer subtypes.

Table S1. PCR Primers

	Sequence 5'-3'
PCR primer for RIF1 splice variant analysis	GTCTCCTTTGGCTTCTCCGT
PCR primer for RIF1 splice variant analysis	GATGTCAACTGGTGCCACAC

Table S2. RT-qPCR Probe Assays

Gene Symbol	Assay ID	RefSeq	Exon Location
RIF1	Hs.PT.58.4552358	NM_001177663(4)	21-22
GAPDH	Hs.PT.39a.22214836	NM_002046(1)	2-2
